# Myocardium area at risk measured with delayed enhancement after scar remodelling compared with T2-weighted cardiac magnetic resonance imaging

**DOI:** 10.1186/1532-429X-13-S1-O112

**Published:** 2011-02-02

**Authors:** Jacob T Lønborg, Niels Vejlstrup, Anders B Mathiasen, Thomas Engstrøm

**Affiliations:** 1 Rigshospitalet, University Hospital of Copenhagen, Copenhagen, Denmark

## Objective

We sought to evaluate the accuracy of the endocardial surface area (ESA) method when applied after scar remodelling (three months after initial infarction) using T2-weigthed imaging as reference.

## Background

Cardiac magnetic resonance (CMR) provides an accurate method to determine myocardial infarct size. The myocardium area at risk (AAR) defined, as the part of the myocardium that is endangered during an acute occlusion of a coronary artery, can retrospectively be determined using T2-weigthed CMR imaging or by measuring the ESA of the infarct on delayed enhancement imaging. Previously, the two methods have been compared in the acute phase within one week after the infarction.

## Method

One hundred and sixty-nine patients with ST-elevation myocardial infarction, treated with primary percutaneous coronary intervention, underwent two CMR scans. The first within one week after initial treatment to determine the AAR with T2-weigthed imaging and a second scan three months after initial treatment to measure final infarct size and AAR with the ESA method. The methods were compared using paired t-test, correlation coefficient and Bland-Altman analysis.

## Results

**Figure 1:**
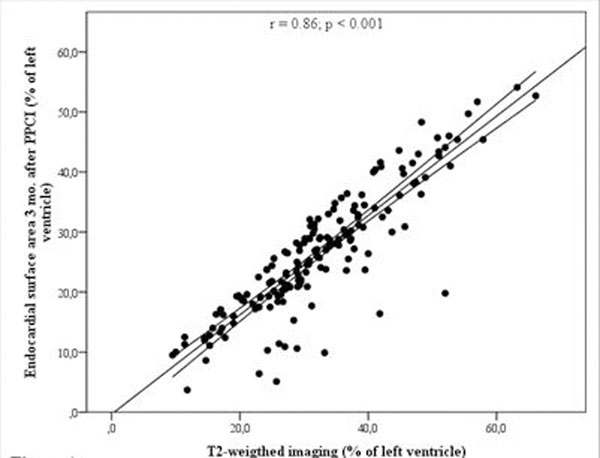


**Figure 2:**
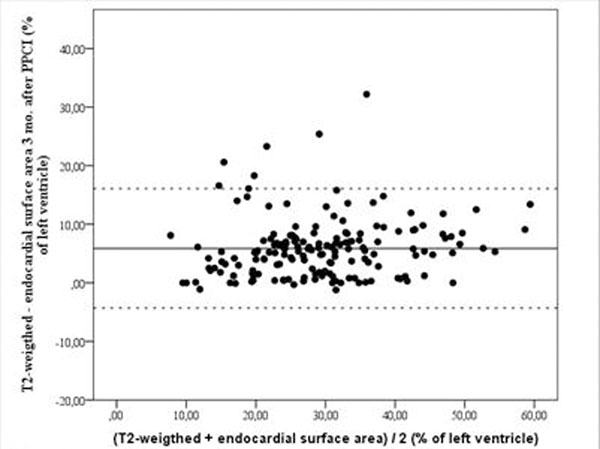
Bottom of Form

The scans were performed 1.7 ± 1.2 days and 89.5 ± 17.3 days after initial treatment, respectively. There was a strong correlation between the two methods (r = 0.86; p < 0.001) (Figure 1). The AAR was significantly higher measured with T2-weigthed imaging than with the ESA methods (32.1 ± 10.9 % of the left ventricle versus 26.2 ± 10.2 % of the left ventricle; p < 0.001). The mean bias was 5.9 ± 5.1 % of the left ventricle. The Bland Altman analysis showed that in all patients except four the T2-weighted measurements were higher than the corresponding ESA measurements (Figure 2.). In the four patients with larger AAR with ESA than with T2-weigthed imaging the difference ranged 0.1 - 1.1 % of the left ventricle.

## Conclusion

In the present study, we report a strong correlation between the ESA method performed after scar remodelling and T2-weigthed imaging in the acute phase as regards to measuring the AAR. Therefore, despite an underestimation of the AAR probably due to early intervention, the ESA method performed after scar remodelling is a valid method to determine AAR.

